# Near-infrared spectroscopy for analysing livestock diet quality: A systematic review

**DOI:** 10.1016/j.heliyon.2024.e40016

**Published:** 2024-11-07

**Authors:** Md Ekramul Hossain, Muhammad Ashad Kabir, Lihong Zheng, David L. Swain, Shawn McGrath, Jonathan Medway

**Affiliations:** a School of Computing, Mathematics and Engineering, Charles Sturt University, Bathurst, NSW 2795, Australia; b Gulbali Institute for Agriculture, Water and Environment, Charles Sturt University, Wagga Wagga, NSW, 2678, Australia; c TerraCipher Pty. Ltd., Alton Downs, QLD 4702, Australia; d Food Agility CRC Ltd, Sydney, NSW 2000, Australia

**Keywords:** Livestock, Diet quality, Near-infrared spectroscopy, Forage, Fecal, Grain feed, Regression model

## Abstract

Near-infrared spectroscopy (NIRS) is a non-invasive and fast technology that has been increasingly used to analyse livestock diet quality. The objective of this study was to conduct a systematic review of the literature to examine the utilisation of NIRS technology for analysing livestock diet quality, with a focus on identifying trends, methodologies, and challenges in recent research. We conducted a systematic search of the literature on five electronic databases and retrieved 718 studies that have been published on the subject. Fifty-four studies were subsequently selected and investigated in depth. These studies were categorised into two groups, namely benchtop and portable, based on the types of NIRS devices utilised, with a majority employing the reflectance spectra mode. Our analysis found that standard normal variate (SNV), detrend (DT), and multiplicative scatter correction (MSC) are the most commonly used spectral data processing methods. The findings indicate that NIRS technology can provide accurate and reliable measurements of key livestock diet quality parameters such as crude protein, fibre, and moisture content. Additionally, we discuss the challenges associated with NIRS technology and provide recommendations for future research directions to further advance the use of NIRS technology in the livestock industry.

## 1 Introduction

Monitoring feed quality can be an important tool in farm management systems to optimise the health and production of livestock. A good understanding of their parameters (e.g., dry or organic matter, crude protein, fat, digestibility, ash, and fibre) can help with farm decision-making and improve farm management. Conventional analytical methods such as wet chemistry need specialised equipment and can be time-consuming and expensive, especially when dealing with many samples [1]. Near-infrared spectroscopy (NIRS) is an alternative to conventional wet chemistry and is a fast, inexpensive, non-destructive, environmentally friendly, reliable, and reproducible analytical approach [2]. NIRS has been used for more than 30 years to measure livestock feed [2], [3] and fecal parameters [4], [5]. These research findings are encouraging but depend on a particular dataset and one or a few analysis techniques. As a result, by compiling their findings systematically and evaluating the pool of results, it will be possible to confirm that NIRS can accurately analyse the characteristics of livestock feed quality.

While significant research has shown the ability of NIRS to measure the parameters of grain feed, fecal, and forage quality [6], [7], [8], [9], rarely are their findings combined to compare, contrast, and critically evaluate. Although several important reviews discussed the use of NIRS for the analysis of livestock diet quality [10], [11], [12], [13], [14], [15], to our understanding, none of these studies considered the rigorous methodology of a ‘systematic review’. This is significant since, unlike conventional literature reviews, a ‘systematic review’ eliminates bias in the inclusion or exclusion of specific literature, which can influence the study's findings [16]. Another issue of the review studies currently available is that they tend to have a narrow focus, often concentrating on a specific aspect of the application of NIRS in livestock production. Recently, Pu et al. [11] reviewed the literature on using portable NIRS in milk, cheese, and dairy powders. A literature review based on the application of NIRS in livestock farming by Chen et al. [13] focused on the analysis of feed protein materials. Kho et al. [14] reviewed the state-of-the-art and future of fecal analysis using NIRS. As these literature review papers focused on the application of NIRS to a particular method of estimating livestock diet quality (e.g., fecal, grain feed, and forage), they lacked a comprehensive systematic review on the use of NIRS for the combination of fecal, grain feed and forage analysis for estimating livestock diet quality. Moreover, the details of the spectra dataset for fecal, grain feed, and forage samples are not discussed. In this context, a systematic scoping review becomes essential, particularly when addressing the challenge of livestock diet quality analysis using NIRS. Also, it is crucial to explore the specifics of the spectra datasets used in relevant studies and thoroughly discuss the current trends observed in applying NIRS in livestock farm management. Furthermore, it is essential to identify future research opportunities and the challenges that need to be addressed in this domain.

Given the recent use and development of rapid and cost-effective NIRS technology in livestock farm management, this study aims to conduct a systematic review on the use of NIRS to analysis livestock diet quality. Although NIRS is used in many areas of livestock farm management, including forage, feed, milk, meat, fecal, and live animal fat analysis, this study specifically focuses on three areas – fecal, grain feed, and forage, for analysing livestock diet quality. In addressing the objectives of the study, several research questions (RQs) were formulated:

• RQ-1: What models are used in livestock diet quality analysis using NIRS?
• RQ-2: What are the differences in technologies and performances for portable and benchtop NIRS devices used in the reviewed articles?
• RQ-3: What are the optimal wavelengths of NIRS found in the reviewed articles for livestock diet quality analysis?
• RQ-4: What parameters are used for analysing livestock diet quality?
• RQ-5: What are the preprocessing methods for analysing spectral data used in the reviewed articles?
• RQ-6: What are the most used models found in the reviewed articles?
• RQ-7: What are the challenges and research opportunities found in the reviewed articles?

In accordance with the defined scope, a total of 54 articles focusing on the analysis of livestock diet quality using NIRS have been carefully selected for this review. These articles are summarised, and the spectra datasets they used are examined. Subsequently, this study analyses the selected articles to identify trends in using NIRS devices for livestock diet quality analysis in recent years. Moreover, the spectral data preprocessing methods, estimated parameters, and prediction models extracted from the reviewed articles are presented. The data extracted from the selected studies are used to address the aforementioned research questions. Finally, the challenges encountered in this field and potential future research directions are discussed.

## 2 Methodology

### 2.1 Review process

This study followed three phases of a review process: planning, conducting and reporting [17]. The planning stage includes identifying research questions for the review. The databases and search terms are determined according to the research questions. Then, the search strings are created using different search terms and used to search various databases to extract relevant articles for the review. In the conducting phase, the primary research studies are found by searching the databases. After that, a set of selection criteria is determined for selecting the eligible studies for the review. The relevant data are then taken from the chosen articles in accordance with the research questions. Finally, the extracted data undergo analysis to address the research questions formulated during the planning phase. The findings are then presented using tables and figures, accompanied by a brief discussion of research challenges and potential opportunities for future investigations.

### 2.2 Search strategy

We followed a search process to get the search results that fulfilled the area of this study. The study used an initial search string with two terms. The search string was (“near infrared spectrometer” AND “livestock diet quality analysis”). A subset of articles was chosen from the search results and subsequently examined to identify similar keywords for the basic search terms. For “near infrared spectrometer”, several synonyms were considered, including “near infrared spectroscopy”, “NIRS”, “handheld spectrometer”, “portable spectrometer” and “near-infrared”. For “livestock”, synonyms considered were “animals” and “live animals”. In terms of quality items, we added several keywords, such as “fecal”, “faecal” “feed”, and “forage”. Thus, the general search string was ((“near infrared spectrometer” OR “near infrared spectroscopy” OR NIRS OR “handheld spectrometer” OR “portable spectrometer” OR “near-infrared”) AND (livestock OR animals OR “live animals” OR “livestock diet quality”) AND (fecal OR faecal OR food OR feed OR forage)). The search terms were used to find articles in five databases in September 2022 – IEEE Xplorer, Science Direct, Scopus, Web of Science, and PubMed. The search strings used for the databases are provided in Table 1. Due to limitations in the maximum number of Boolean connectors (AND/OR) allowed by the Science Direct database, certain terms were excluded from the search string for this database. Moreover, this study restricted the search to publications from 2012 to 2022. Following the execution of the aforementioned search strings, 718 articles were obtained.

**Table 1:** Search strings employed for querying the selected databases.

Database name	Search string
Scopus	ABS ((“near infrared spectrometer” OR “near infrared spectroscopy” OR NIRS OR “handheld spectrometer” OR “portable spectrometer” OR “near-infrared”) AND (livestock OR animals OR “live animals” OR “livestock diet quality”) AND (fecal OR faecal OR food OR feed OR forage)). The search was conducted specifically within the abstract (ABS).
IEEE Xplore	((“near infrared spectrometer” OR “near infrared spectroscopy” OR NIRS OR “handheld spectrometer” OR “portable spectrometer” OR “near infrared” OR “near-infrared”) AND (livestock OR animals OR “live animals” OR “livestock diet quality”) AND (fecal OR faecal OR food OR feed OR forage)) (anywhere).
Science Direct	((“near infrared spectrometer” OR “near infrared spectroscopy” OR NIRS) AND (livestock OR animals OR “livestock diet quality”) AND (fecal OR forage OR feed)). The search was conducted within the title, abstract, and keywords.
Web of Science	X=((“near infrared spectrometer” OR “near infrared spectroscopy” OR NIRS OR “handheld spectrometer” OR “portable spectrometer” OR “near infrared” OR “near-infrared”) AND (livestock OR animals OR “live animals” OR “livestock diet quality”) AND (fecal OR faecal OR food OR feed OR forage)). The search string format for Web of Science is - AB(X) OR AK(X) OR TI(X). The search was conducted within the title (TI), abstract (AB), and author keywords (AK).
PubMed	((“near infrared spectrometer” OR “near infrared spectroscopy” OR NIRS OR “handheld spectrometer” OR “portable spectrometer” OR “near infrared” OR “near-infrared”) AND (livestock OR animals OR “live animals” OR “livestock diet quality”) AND (fecal OR faecal OR food OR feed OR forage)). The abstract and title were used as the search fields for conducting the search.

### 2.3 Selection criteria

The publications capable of addressing the research questions were selected using specific criteria. In this review, the inclusion and exclusion criteria were established based on the research objectives. The exclusion criteria encompassed the following aspects: (i) publications that did not use NIRS for livestock diet quality analysis, (ii) survey or review papers, and (iii) publications not written in English. On the other hand, the inclusion criteria specified that the publications must use NIRS for analysing fecal, forage, and grain parameters.

**Figure 1:**
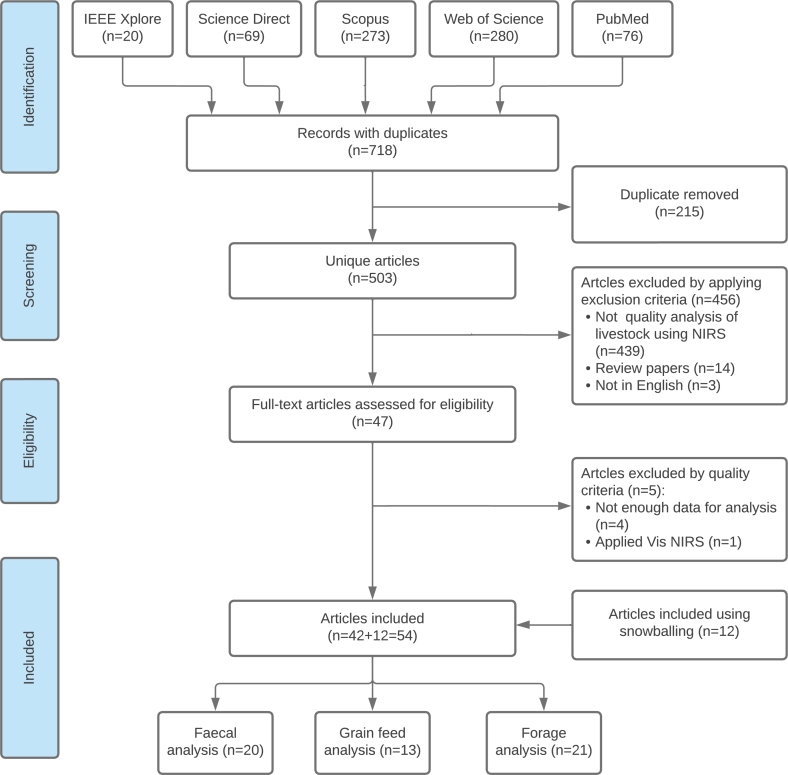
Flowchart illustrating the article selection process for the review.

After removing 215 duplicate records, the remaining records (n=503) were subjected to the selection criteria. Consequently, a total of 47 full-text articles underwent eligibility assessment. During the evaluation of eligibility and quality, an additional five full-text articles were excluded for various reasons, including inadequate data for analysis and the use of Visible NIRS. The study followed a quality assessment process [18] and used backward and forward snowballing techniques [19] to identify additional relevant articles, resulting in the discovery of 12 more articles. Consequently, a total of 54 articles were ultimately chosen for this review. The entire article selection process is illustrated in Fig. 1 using PRISMA. Furthermore, searches were conducted in electronic databases such as the Association for Computing Machinery (ACM), Springer, and others, using specific search strings. However, the related articles retrieved from these searches were duplicates of the ones already included in our selected databases and so were not detailed in the PRISMA report.

### 2.4 Data analysis

Each qualified article was fully read, and the relevant data attributes were taken out to answer the RQs. All the data that was extracted from the qualified articles was entered into a spreadsheet. The rows in the spreadsheet correspond to the articles reviewed, while the columns represent the various study data aspects. Each article's summary in the spreadsheet includes the research objective, dataset information, NIRS device details, spectral data preprocessing methods, experiment location, published year, models, performance, and problems. Then, the retrieved data are categorised in accordance with the study's objectives. The findings of the study are described in Section 4.

## 3 Near-infrared spectroscopy technology

Near-infrared spectroscopy (NIRS) examines the interaction between chemical substances and electromagnetic radiation [20]. NIRS operates in the wavelength range between 780 and 2500 nm, which lies between the visible infrared (VIR) and mid-infrared (MIR) regions. This range is characterised by overtones and combination transitions of the fundamental vibrations of molecules [21]. As NIR radiation passes through a sample, it is selectively absorbed at these specific wavelengths based on the vibration frequencies of the molecules present, generating a spectrum that reflects the sample's composition. The frequencies of absorbed radiation provide the unique characteristics of the sample.

The NIRS instrument consists of three basic components: a light source, a detector, and a wavelength selector. The first NIRS instruments were designed for laboratory use and were very expensive and sophisticated. Only trained staff could use them, although they offered accurate analytical results. The development of portable instruments has been made possible by the evolution of the instruments, which have significantly decreased in size and weight. The performance of the recent instruments has increased due to their speedy spectrum acquisition and improvements in signal processing and transmission techniques. In addition, the hardware and software of NIRS instruments have been improved in recent years [10].

**Figure 2:**
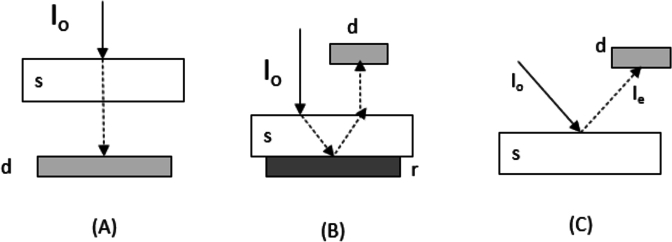
Different modes of NIRS measurements. (A) transmittance, (B) transflectance, and (C) reflectance. *I*_*o*_ - Incident beam, *I*_*e*_ - emerging beam, s - sample, d - detector, and r - reflecting surface.

NIRS measurements can be taken in the transmittance, reflectance, or transflection modes. In the transmittance mode, the light crosses the entire surface of the sample, and then the information is collected based on the entire object volume. The reflectance mode is used to get information about the surface of the sample. The transflectance mode combines the two aforementioned modes and is best suited for liquid samples [10]. Fig. 2(A), 2(B), and 2(C) show the transmittance, reflectance, and transflection modes of NIRS measurements, respectively.

Calibration is important for using NIRS techniques [2]. NIRS calibration involves analysing the electromagnetic spectrum and the corresponding physical and chemical composition information (reference data) using various statistical methods. Linear regression (LR), partial least squares regression (PLSR), and principal component regression (PCR) are among the most commonly used statistical approaches for NIRS calibration. These methods enable the establishment of a quantitative relationship between the spectral data and the target composition, facilitating accurate predictions and analysis. It is difficult to obtain a robust and accurate NIRS calibration model as it requires a large volume of data to include all types of physical and chemical properties of the sample. The performance of the NIRS calibration model is evaluated in terms of accuracy, coefficient of determination ($R^{2}$), calibration standard error (SEC), and the ratio of performance to deviation (RPD). These chemometric methods provide a mathematical model using the spectra data and reference data (physical and chemical information of the sample). Then, the model is used to measure the parameters of the unknown samples.

## 4 Review summary

**Figure 3:**
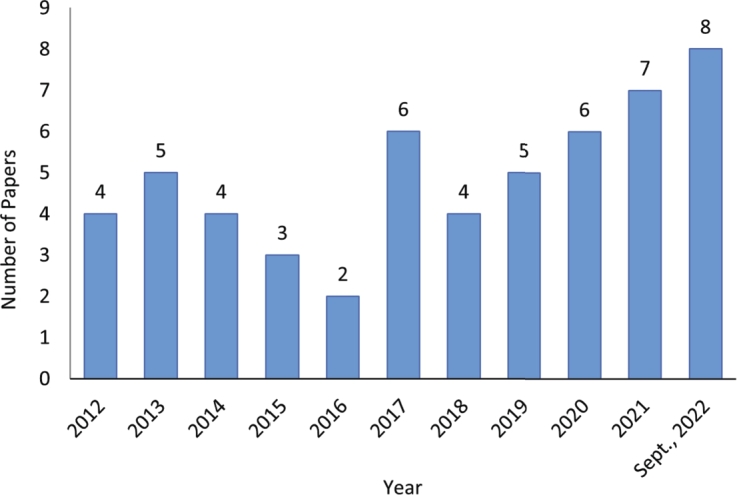
The distribution of the selected papers in terms of publication year.

**Figure 4:**
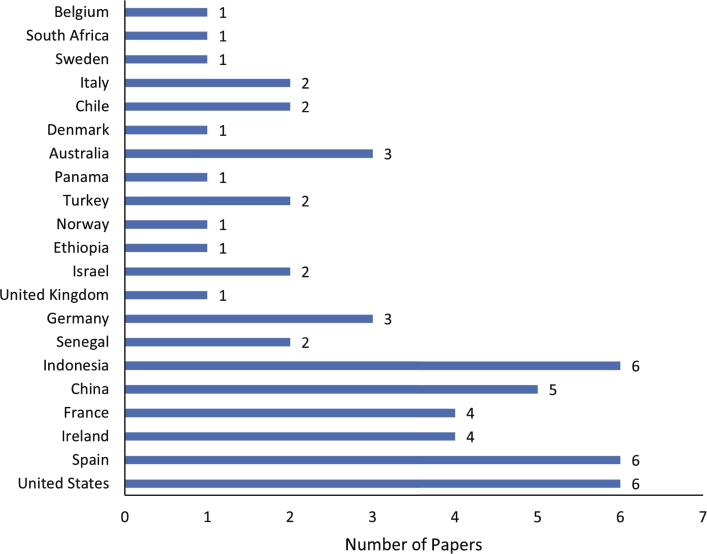
Distribution of countries where the experiments were carried out for the reviewed papers.

In this review, we selected 54 papers for review after applying the selection criteria. The number of these papers is distributed by published year and shown in Fig. 3. It is observed that more research has used NIRS to analysis livestock diet quality in recent years. This might be due to the need to analyse the livestock diet quality in a fast and cost-effective way. Fig. 4 presents the distribution of countries where the experiments of the reviewed articles were conducted. It is observed that the research experiments were conducted in 21 countries, mainly the United States (6), Spain (6), and Indonesia (6), followed by China (5), Ireland (4), France (4), and Australia (3). The concentration of NIRS research in countries like the United States, Spain, Indonesia, China, Ireland, France, and Australia can be attributed to their robust agricultural sectors and significant investments in livestock production [22], [23]. These nations prioritise optimising feed quality and efficiency to enhance livestock productivity and sustainability, reflecting their strong research infrastructure in agricultural sciences. The extensive livestock industries in the United States and China [24], [25], the focus on feed efficiency in Spain and France [26], and the commitment to sustainable farming practices in Ireland and Australia [27], [28], all contribute to their leadership in utilising advanced technologies like NIRS. In Indonesia, the use of NIRS is driven by the need to optimise feed resources in diverse and challenging agricultural environments [29].

**Figure 5:**
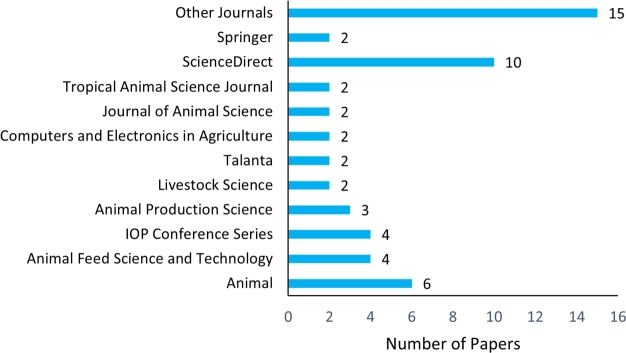
Distribution of journals and conferences for the selected papers related to using NIRS for livestock diet quality (published 2012-2022).

Fig. 5 shows the distribution of journals and conferences for the reviewed studies. The 54 publications examined for this study were distributed among 37 peer-reviewed journals and conferences. In the figure, we categorise 10 journals of ScienceDirect publisher as “ScienceDirect”. Similarly, two journals published by Springer are referred to as “Springer”. The figure indicates that Animal, Animal Feed Science and Technology, IOP Conference Series, and Animal Production Science Journal are the four top outlets that published the highest number of papers related to livestock diet quality analysis using NIRS. The other five outlets, Livestock Science, Computer and Electronic Agriculture, Journal of Animal Science, Talanta, and Topical Animal Science Journal, published two papers in this research area.

### 4.1 Summary of the selected papers

In this review, we categorised the reviewed papers into three groups in terms of livestock diet quality according to the scope of this study. They are fecal analysis, forage analysis, and grain feed analysis. Fecal analysis can have an important application for assessing the health and well-being of livestock. The analysis of fecal samples from livestock can provide valuable information about the animal's nutritional status, digestive function, and overall health. Table 2 summarises the reviewed studies relevant to the fecal analysis for estimating livestock diet quality. The summary includes the livestock species, dataset sample size, experiment location, parameter, spectra prepossessing method, NIRS device, model, and performance. The animal column indicates the name of livestock species and their total numbers used in the experiment. The sample size represents the total number of fecal samples, along with the number of samples used for calibration and prediction, if this information is available in the reviewed article. It is observed that half of the fecal analysis related studies used cattle in their experiment. This is partially because of increased cattle farming for the increased rate of dairy and beef production [30]. Other livestock, including horses, pigs, deer, goats, and sheep are also significantly used in the experiment for fecal analysis using NIRS.

**Table 2:** Summary of the articles related to fecal analysis for estimating livestock diet quality.

Reference	Animal	Sample size (Cal/Pred)	Exp location	Parameter	NIRS instrument	Preprocess method	Model	Performance
[4]	C(12)	680	USA	–	Foss NIRS 6500	SNV, DT	PCA	*p* < 0.005
[6]	H (29)	116 (87/29)	Ireland	OM, NDF, ADF, ADL	Foss NIRS DS2500	SNV, DT, MSC	MPLSR	0.92 (*R*^2^), 3.54 (RPD)
[34]	C (457)	1074 (806/268)	Ireland	DMI	Foss NIRS 6500SY II	Derivative	PLSR	0.68 (*R*^2^), 1.52 (RMSE)
[35]	C, S, G	848	Senegal	DM, dOM	Lab Spec4	–	–	–
[5]	S (24)	96	Spain	Ash, NDF, CP, NFC	Foss NIRS 6500SY II	SNV, MSC	MPLSR	0.98 (*R*^2^), 41.9 (SECV)
[36]	P (20)	196 (146/50)	France	dDM, dOM, ADE	Foss NIRS 6500	SNV, MSC	MPLSR	0.67 (*R*^2^), 1.12 (SEP)
[37]	C	–	France	PEG, Yb	Foss NIRS 6500	SNV, DT	MPLSR	0.98 (*R*^2^), 3.45 (SECV)
[38]	S	527	France	DMI, PEG	Foss NIRS 6500	SNV, DT	MPLSR	0.99 (*R*^2^), 9.1 (RPD)
[39]	G	147	Israel	CP, ADF NDF, IVDMD	Foss NIRS 5000	SNV, DT	MPLSR	0.93 (*R*^2^), 0.87 (SECV)
[40]	D(73)	100	Spain	ADF, NDF	Foss NIRS DS2500	SNV, DT	GLMM	0.98 (*R*^2^)
[41]	P	198 (158/40)	Norway	dOM, CP	Foss NIRS 6500	SNV, DT	LR	0.94 (*R*^2^), 5.5 (SEP)
[42]	C (210)	12	USA	dOM, CP	NIRS Scanner 4250	–	LR	0.95 (*R*^2^), 0.91 (SECV)
[43]	C, G,S	512 (384/128)	USA	N, P	Foss NIRS 6500	SNV, DT	PLSR	0.97 (*R*^2^), 0.13 (SECV)
[44]	C (20)	405	Australia	DMD	Foss NIRS 6500	–	LR	*p* < 0.01
[45]	P	757 (607/150)	Denmark	CP, Amino acid	Foss NIRS DS2500	SNV	MPLSR	0.97 (*R*^2^), 0.71 (SEP)
[46]	C	125	Israel	Ash, NDF, CP, IVDMD	Foss NIRS 5000	SNV	MPLSR	0.89 (*R*^2^), 31 (SECV)
[27]	S	124 (99/24)	Australia	OMI, dOM	FT-NIR MPA500	–	PLSR	0.96 (*R*^2^)
[47]	B	80	SA	N, P, NDVI	Foss NIRS 5000	–	GLM	0.98 (*R*^2^)
[48]	C (44)	–	Belgium	dOM, DMI	Foss NIRS 5000	–	LR	0.88 (*R*^2^)
[49]	C	130	Italy	NDF	Foss NIRS DS2500	–	PLSR	0.59 (*R*^2^), 1.52 (RPD)

Cal=Calibration, Pred= Prediction, C=Cattle, H=Horse, G=Goat, S=Sheep, D=Deer, P=Pig, B=Buffalo,

MSC= Multiplicative scatter correction, SNV=Standard normal variate, DT=Detrend, Yb=Ytterbium,

OM=Organic matter, ADF=Acid detergent fibre, ADL=Acid detergent lignin, NDF=Neutral detergent fibre, DMI=Dry matter intake,

dOM-Digestibility of organic matter, dDM=Digestibility of dry matter, CP=Crude protein, NFC=Non fibrous carbohydrates,

ADE=Apparent digestible energy, NVDI=Normalised difference vegetation index, PEG=Polyethylene glycol, DM=Dry matter,

ATTD= Apparent total-tract digestibility, IVDMD=In vitro drymatter digestibility, N=Nitrogen, P=Phosphorus,

MPLSR=Modified partial least square regression, PLSR=Partial least squareregression, PCA=Principal component analysis,

PCR=Principal component regression, GLMM=generalised linear mixed model, GLM=generalised linear model, LR= linear regression.

Forage analysis is the process of analysing the nutrient content of feedstuffs, including hay, silage, and pasture that are fed to or grazed by livestock. The analysis provides valuable information about the nutritional quality of the feed, which is essential for ensuring the health and productivity of livestock. Table 3 summarises the 21 articles related to forage analysis for livestock diet quality. The summary is represented by forage sample size with split ratio, experiment location, parameter, NIRS devices, spectra prepossessing method, model, and performance. It is observed that significant research has been completed for analysing forage samples using portable NIRS devices, as portable devices have become available and researchers have tested the application and accuracy of this technology in recent years [31], [32].

**Table 3:** Summary of the articles related to forage analysis for livestock diet quality.

Reference	Sample size (Cal/Pred)	Exp location	Parameter	NIRS instrument	Preprocess method	Model	Performance
[31]	60 (40/20)	France	CP, TS	LabSpec 4 SCiO	SNV, DT, MSC	SO-PLSR	0.91 (*R*^2^), 0.69 (SEP)
[7]	809(647/162)	Spain	DM, CP, NDF	Foss NIRS 5000	SNV, DT, MSC	MPLSR	0.98 (*R*^2^), 2.2 (SEP)
[32]	–	Germany	Ash, NDF	FieldSpec 3, XDS RCA	SNV	MPLSR	0.98 (*R*^2^), 0.9 (SECV)
[50]	564 (513/51)	Spain	DM, CP, NDF	Foss NIRS 6500	SNV	PLSR	0.41 (RMSEP)
[51]	1128	USA	CP, ADF,ADL, NDF, IVTD	Foss NIRS 6500 SCiO	SNV	PLSR	0.96 (*R*^2^), 1.20 (SEP)
[3]	190 (160/30)	Ethiopia	Ash, CP, DM, NDF, ADF	Foss NIRS 5000	SNV	LR	0.68 (*R*^2^), 0.9 (SEP)
[52]	216 (151/65)	Turkey	Ash, CP, DM, NDF, ADF	Foss XDS	–	MPLSR	0.95 (*R*^2^), 1.02 (SEP)
[53]	310 (233/77)	USA	CP, IVTD, NDF, ADF	Foss XDS	SNV, MSC, DT	PLSR	0.98 (*R*^2^), 8.1 (SEP)
[54]	153 (123/30)	Panama	CP, NDF, ADF	InfraXactTM	SNV	PLSR	0.90 (*R*^2^), 1.8 (REP)
[55]	123 (93/30)	China	CP, NDF, ADF, WSC	MPA FT-NIRS	SNV, MSC	PLSR	0.99 (*R*^2^), 9.37 (RPD)
[56]	353 (253/100)	Turkey	CP, NDF, ADF, CF	XDS-NIRS	MSC	MPLSR	0.93 (*R*^2^)
[57]	4385 (3885/500)	Australia	CP, NDF, ADF, dMDCP,	Spectrastar 2500X	SNV	PLSR	2.2 (RPD), 1.06 (SECV)
[9]	915	Chile	DM, CP, NDF, dOM, IVDMD	MPA FT-NIRS	VN, MSC	PLSR	0.93 (*R*^2^), 1.13 (RMSEP)
[58]	1812 (1615/197)	Ireland	DM, CP	Foss NIRS 6500	SNV, DT, MSC	MPLSR	0.86 (*R*^2^), 2.60 (RPD)
[59]	90	Spain	CP, NDF, ADF	NIRscan Nano	SNV	PLSR	0.74 (*R*^2^), 2.11 (SEC)
[60]	151	China	CP, NDF, ADF	DA7200	–	FLQR	0.67 (RMSE)
[61]	410	USA	CP, NDF, ADF, IVTD	SpectraStar 2600 XT-R	–	SVM	0.95 (*R*^2^), 2.23 (RMSE)
[62]	100	Italy	CP, NDF, ADF, Ash	NIRS Antaris II	MSC	PLSR	0.92 (*R*^2^)
[63]	132	Sweden	CP, WSC, NDF, dOM	Foss NIRS 6500	SNV, MSC	PLSR	0.93 (*R*^2^), 13.6 (RMSE)
[64]	297 (115/52)	Chile	CP, DM, IVD, ME	FT-NIR MPA	–	PLSR	0.99 (*R*^2^), 0.46 (RMSE)
[65]	100 (80/20)	Indonesia	CP, DM, NDF, ADF, ADL	NIRFlex N-500	–	PLSR	0.89 (*R*^2^)

WSC=Water soluble carbohydrate, TS=Total sugar content, SO-PLSR=Sequential and orthogonalised - partial least squares,

FLQR=functional linear quantile regression.

Feed analysis provides valuable information about the feed's composition, including protein, fibre, energy, minerals, and vitamins. This information is critical in ensuring that livestock receives the appropriate amount and balance of nutrients required for optimal health and productivity. The necessary information extracted from the 13 articles related to grain feed analysis is summarised in Table 4. The summary includes the same information as shown in Table 3. In this review, numerous reviewed papers used multiple regression models. In such instances, the performance of the most effective model is reported in Table 2, Table 3, Table 4.

**Table 4:** Summary of the articles related to grain feed analysis for livestock diet quality.

Reference	Sample size (Cal/Pred)	Exp location	Parameter	NIRS instrument	Preprocess method	Model	Performance
[66]	99 (66/33)	Spain	n-alkanes, LCOH	Nicolet iS50	SNV	PLSR	0.90 (*R*^2^), 7.59 (RPD)
[33]	193 (146/47)	China	Ash	Nicolet Antaris II	SNV	ABDE	0.93 (*R*^2^), 1.12 (RMSEP)
[67]	25	Indonesia	IVOMD, NDF, IVDMD, ADF	Foss NIRS 5000	BSC, SNV, DT	PLSR	0.87 (*R*^2^), 0.48 (RMSE)
[68]	100	Senegal	CP, CF, Starch	Phazir 1624 MicroNIR 1700	SNV, MSC	PLSR	0.91 (*R*^2^)
[69]	–	Germany	Melamine	Nicolet Antaris II	SNV	PLSR	0.99 (*R*^2^), 0.13 (RMSEP)
[70]	34	UK, Ireland	Moisture	Nicolet Antaris II	SG, SNV	PLSR	0.99 (*R*^2^), 0.5 (RMSEP)
[8]	108 (72/36)	China	Moisture	NIRS BIO-JHWGPY	SG	PLSR	0.99 (*R*^2^), 0.006 (RMSEP)
[71]	30	Indonesia	DM, ash, CF	–	SNV	PLSR	0.7 (*R*^2^), 1.5 (RPD)
[72]	30	Indonesia	DM, CP, CF, Ash	–	PCA	–	–
[29]	25	Indonesia	IVDMD, IVOMD	–	DT, SNV	PCR	0.93 (*R*^2^), 2.35 (RPD)
[73]	25	Indonesia	NDF, IVDMD, ADF, IVOMD	–	BSC, DT, SNV	PCR	0.93 (*R*^2^), 2.78 (RPD)
[74]	50	Germany	TIA	TANGO FT-NIRS	MSC, derivative	PLSR	0.93 (*R*^2^), 2.05 (RMSECV)
[75]	33 (24/9)	China	DE, ME	ATRIX I FT-NIRS	MSC, SNV	PLSR	0.90 (*R*^2^), 2.35 (RPD)

IVOMD=In vitro organic matter digestibility.

In terms of NIRS instrumentation, it is worth mentioning that most of the papers used a lab-based benchtop FOSS NIRS device for fecal analysis. Foss NIRS is popular for fecal analysis due to its ability to handle complex and varied biological samples efficiently. It is a rapid, non-destructive method [4], [5]. However, for grain feed analysis, several challenges including sample preparation and variability requirements, make it more difficult to apply NIRS effectively [33].

### 4.2 Spectrometers used in the selected papers

The selection of the right spectrometer instruments is essential for effective NIR application, and these instruments have advanced greatly in response to the demand for robust and quick analysis and adaptability to various sample conditions. Table 5 presents the various NIR spectrometers used in the reviewed articles for analysing livestock diet quality. This study divided the spectrometers into two groups: benchtop (lab-based) and portable (field-based). According to the table, NIRS application in livestock diet quality analysis is not limited to a specific spectrometer type or brand. However, the table indicates that the top 2 most used benchtop spectrometers for this type of application are FOSS NIRS 6500 and FOSS NIRS 5000. The other widely used laboratory-based spectrometers are FOSS NIRS DS2500, Antaris II FT-NIR, MPA FT-NIRS, and XDS RCA. In terms of field-based spectrometers, DLP NIRscan Nano, ASD LabSpec 4, SCiO, and PHazir 1624 are the most used instruments for analysing livestock diet quality, as shown in Table 5.

**Table 5:** Overview of NIR spectrometers used in the reviewed articles.

Type	Name	Company & Country	Wavelength range (nm)	Spectra mode	Paper reference	Count
Benchtop	Zeiss Corona 45	Carl Zeiss Inc., Germany	302-1711	R	[50]	1
	Foss NIRS 6500	Foss Analytical, Denmark	400-2498	R	[4], [36], [37], [50], [38], [51], [41], [43], [53], [44], [58], [63]	12
	XDS RCA	Foss Analytical, Denmark	400-2500	R	[32], [52], [56]	3
	NIR Scanner 4250	Pacific Scientific, USA	400-2500	R	[42]	1
	ATR-FT NIR	Bruker, Germany	500-2500	R	[75]	1
	Spectrastar 2600 XT-R	Unity Scientific, USA	680-2600	R	[61]	1
	InfraXact	Foss Analytical, Denmark	800-1850	R	[54]	1
	MPA FT-NIRS	Bruker, Germany	800-2500	R	[55], [9], [27], [64]	4
	NIRFlex N-500	BUCHI, Switzerland	800-2500	R	[65]	1
	Antaris II FT-NIR	Thermo Fisher Scientific, USA	830-2630	R, D	[69], [70], [55], [62]	4
	TANGO FT-NIR	Bruker, Germany	870-2500	R	[74]	1
	DA7200	Perten Corporation, Sweden	950-1650	R	[60]	1
	Nicolet iS50 FTIRS	Thermo Fisher Scientific, USA	1000-2500	D	[66]	1
	Nicolet Antaris II	Thermo Fisher Scientific, USA	1000-2500	R	[67]	1
	NM	NM	1000-2500	D	[71], [72], [29], [73]	4
	Foss NIRS DS2500	Foss Analytical, Denmark	1100-2500	R	[6], [40], [45], [49]	4
	Foss NIRS 5000	Foss Analytical, Denmark	1100-2500	R	[33], [39], [3], [46], [47], [48]	6
	Foss NIRS 6500 SY II	Foss Analytical, Denmark	1100-2498	R	[34], [5]	2
	Spectrastar 2500X	Unity Scientific, USA	–	R	[57]	1

Portable	ASD LabSpec 4	Malvern Panalytical, USA	350-2500	R	[31], [35]	2
	ASD FieldSpec 3	Malvern Panalytical, USA	420-2400	R	[32]	1
	F750	FelixInstrument, USA	450-1140	R	[31]	1
	SCiO	Consumer Physics, Israel	740-1070	R	[31], [51]	2
	NIR Instrument	BIO-JHWGPY, China	900-1700	R	[8]	1
	mNIR2200	Viavi, USA	901-1701	R	[31]	1
	mNIR1700	Viavi, USA	908-1676	R	[31], [68]	2
	Aurora Nir	Grainit S.r.l., Italy	950-1650	R	[51]	1
	NIR-S-G1	Innospectra, Taiwan	950-1650	R	[51]	1
	DLP NIRscan Nano	Texas Instruments, USA	1150-2150	R	[31], [53], [55]	3
	PHazir 1624	Polychromix, USA	1600-2400	R	[68], [53]	2
	NIRONE 2.2	Spectral Engines, Finland	1750-2150	R	[31]	1

R=Reflectance, D= Diffuse reflectance, NM=Not Mentioned.

The period from 2012 to 2022 witnessed significant advancements in NIRS technology, particularly in the development of both portable and benchtop NIRS devices. Over this decade, we observed a steady increase in the number of published research papers (Fig. 3), indicating a growing interest and application of NIRS for analysing livestock diet quality. Benchtop NIRS devices have traditionally been used in laboratory settings and are characterised by their high accuracy, stability, and the ability to analyse a wide range of samples with minimal interference [4], [36], [37], [64]. These devices have benefited from continuous improvements in spectral resolution, data processing capabilities, and the development of more sophisticated calibration models. Throughout the decade, benchtop NIRS remained the gold standard for in-depth and high-precision analyses [66], [55], [9]. The most notable technological advancement during this period was the development and increased adoption of portable NIRS devices. Portable NIRS devices have become increasingly compact, user-friendly, and capable of providing real-time analysis in field conditions [31], [35]. Improvements in battery life, wireless connectivity, and integration with mobile platforms have made these devices more accessible and practical for on-site analysis, even in remote locations [51], [53]. While portable NIRS devices historically lagged behind benchtop models in terms of accuracy and spectral range, the gap has been narrowing due to technological advancements, such as better calibration methods and enhanced data processing algorithms.

A total of eight instruments out of 31 used a wavelength of 300–2500 nm for both VIR and NIR. The rest of the instruments used the NIR spectrum region (780–2500 nm) for the livestock diet quality analysis. Some articles mentioned only the optimal wavelength range (1000–2500 nm) for forage, fecal, and grain feed analysis [66], [6], [31], [67], [40]. It is also observed that most of the NIRS instruments used the reflectance spectra mode, while few used the diffuse reflectance [66], [71], [72]. The preference for the reflectance mode is due to its simplicity and reliability, as it primarily measures light directly reflected off the sample surface, leading to stronger and more consistent signals. In contrast, diffuse reflectance involves light scattering within the sample, which can introduce variability due to factors like sample heterogeneity and particle size, making it more complex to interpret [20]. These challenges make diffuse reflectance less favourable for routine analyses, particularly in the context of livestock diet quality, where consistency and ease of use are critical. However, it is important to emphasise that for solid and powdered samples in agricultural applications, diffuse reflectance spectroscopy (DRS) is the primary method of reflectance used [76]. Accessories such as integrating spheres and fibre optic probes are typically employed to enhance the collection of diffusely scattered light, ensuring more accurate and representative data. The integrating sphere, for example, captures light reflected in multiple directions, providing a comprehensive assessment of the sample's optical properties, while fibre optic probes allow for in situ or portable measurements, enhancing the versatility of DRS in field studies [77]. By leveraging DRS in combination with appropriate accessories and preprocessing methods, NIRS enables robust analysis of complex samples, providing valuable insights into the diet quality of livestock through the characterisation of forages, grains, and fecal matter.

**Figure 6:**
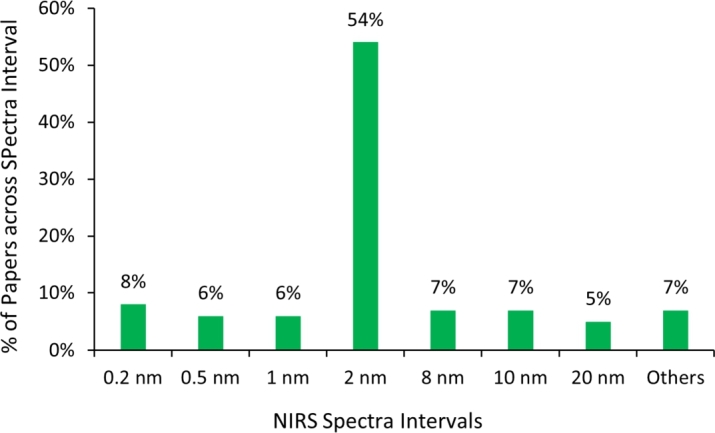
Spectra intervals used in the reviewed papers.

Fig. 6 illustrates the spectral intervals utilised in the reviewed papers. Notably, approximately 54% of these studies employed a 2 nm spectral interval during the collection of spectra using NIRS instruments. This prevalence can be attributed to the specific characteristics of the NIRS instruments utilised in those studies.

### 4.3 Spectral data preprocessing methods

**Figure 7:**
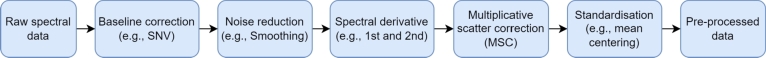
Flowchart of the NIRS spectral data preprocessing steps.

Preprocessing of NIR spectra data is an essential part of chemometrics modelling. The preprocessing treatment aims to eliminate noise or physical interference from the spectra to enhance the model's performance. Fig. 7 shows the flowchart of the NIRS spectral data preprocessing steps. The sequence was selected based on established best practices in NIRS data processing, where each step builds upon the previous one to progressively reduce noise and correct for various physical effects. Baseline Correction is typically the first step to remove baseline shifts that could distort the spectral data and interfere with subsequent analysis. After baseline correction, noise reduction is applied to smooth the spectra and eliminate high-frequency noise that could affect derivative and calibration steps. The derivative is then calculated to enhance spectral resolution and separate overlapping peaks, making the features of interest more distinct. Multiplicative Scatter Correction (MSC) step follows to correct for scatter effects caused by particle size or other sample characteristics, ensuring more accurate analysis. Lastly, standardisation ensures that the processed spectra are comparable across different instruments or measurement conditions, facilitating model robustness.

**Figure 8:**
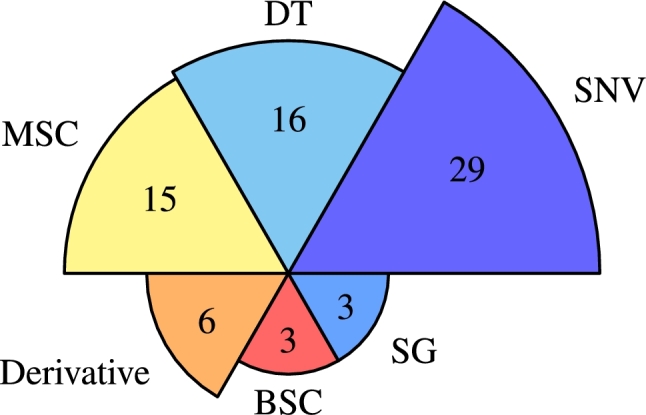
Spectral data preprocessing methods used in the reviewed papers. SNV: Standard Normal Variate, MSC: Multiplicative Scatter Correction, DT: Detrend, BSC: Baseline Shift Correction, SG: Savitzky-Golay.

The reviewed studies used various preprocessing approaches and mathematical pretreatments to smooth the spectral data, as shown in Fig. 8. The preprocessing methods included standard normal variate (SNV), first and second derivatives, detrend (DT), multiplicative scatter correction (MSC), Savitzky-Golay (SG), and baseline shift correction (BSC). The figure indicates that SNV, MSC, and DT are the most common spectra data preprocessing methods used in the reviewed studies. Most studies used multiple preprocessing methods and compared the results, while some used only one method (shown in Table 2, Table 3, Table 4). It is worth mentioning that 12 studies did not mention any spectral data preprocessing methods.

In reviewing the studies, we observed that the choice of preprocessing methods often depends on the specific sample type and the target parameters being analysed. SNV was the most commonly used preprocessing method across fecal, forage, and grain feed samples. SNV is particularly effective in reducing scattering effects caused by particle size variation, which makes it suitable for complex biological samples such as faeces [5], [36], [45], forage [7], [31], [54], and grain feed [66], [67], [68].

DT was often used in combination with SNV to correct baseline shifts in spectra, particularly in heterogeneous samples like forage and fecal materials. DT was commonly applied when analysing parameters such as ADF, NDF, and CP in forage [58], [59] and dOM, CP, and DMD in fecal analysis [40], [41].

MSC was employed to adjust for scatter effects and was effective in cases where scatter dominated the spectral data, especially in forage and grain feed samples. This method was frequently used for parameters such as starch, DM, and CP in grain feed [66], [74], and for parameters like CP, NDF, and ADF in forage samples [57], [55].

In grain feed analysis, derivative methods were sometimes combined with SNV and MSC to further correct baseline shifts and reduce noise. This combination was particularly useful for challenging parameters like IVDMD and IVOMD [67], [73].

The selection of preprocessing methods is largely dependent on the sample type and the complexity of the target parameters. SNV is versatile and widely applied, while DT and MSC provide additional corrections for scatter and baseline shifts, making them particularly effective for forage and grain feed samples with heterogeneous content. By carefully selecting preprocessing methods, the studies were able to enhance model performance and improve the accuracy of NIRS-based predictions in livestock diet quality analysis.

### 4.4 Parameters for livestock diet quality analysis

**Figure 9:**
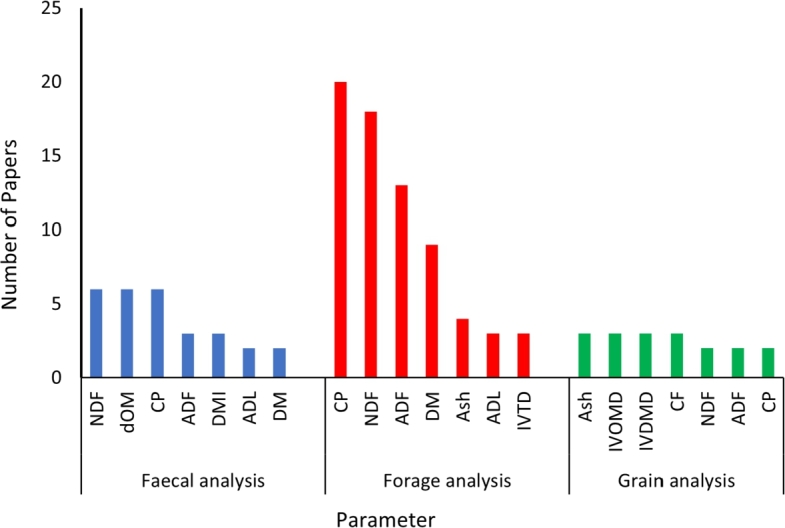
Parameters for fecal, forage, and grain analysis used in the reviewed papers.

Fig. 9 presents the fecal, forage, and grain feed analysis parameters extracted from the selected papers. These forage, fecal, and grain feed parameters were predicted by NIRS in the reviewed articles. Most of the 54 articles used more than one forage, fecal, and feed parameter for analysing livestock diet quality. In terms of fecal analysis, it is observed that the three most commonly used parameters were neutral detergent fibre (NDF), digestibility of organic matter (dOM), and crude protein (CP). The other predicted fecal parameters for estimating livestock diet quality were acid detergent fibre (ADF), dry matter intake (DMI), and acid detergent lignin (ADL). Similarly, CP, NDF, and ADF are the most common forage parameters for analysing livestock diet quality, followed by dry matter (DM), ash, and ADL. The figure indicates that the most commonly predicted parameters for grain analysis were ash, in vitro organic matter digestibility (IVOMD), and in vitro dry matter digestibility (IVDMD). It is worth mentioning that NDF, ADF, and CP are the common parameters in the three cases (fecal, forage, and grain feed analysis).

### 4.5 Regression models

**Figure 10:**
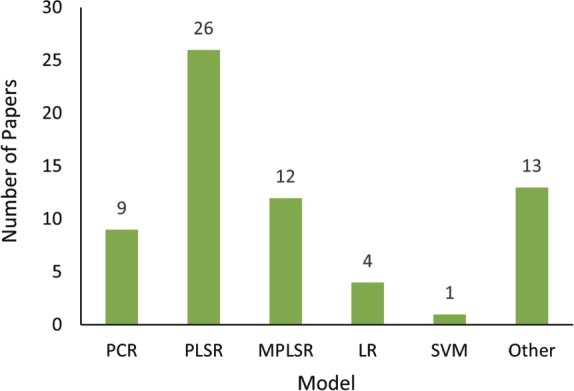
Regression models used in the reviewed papers.

Several regression models have been used to extract fecal, forage, and grain feed properties from the NIR spectra. The input of these models is the reflectance or diffuse reflectance of the samples at different wavelengths. These models are trained using standard laboratory measurements of sample properties. The performance of these models is calculated by comparing their output with the laboratory measurements through evaluation metrics, including coefficient of determination ($R^{2}$), root mean square error (RMSE), calibration standard error (SEC), and the ratio of performance to deviation (RPD). Fig. 10 reports the various regression models used in the reviewed articles for analysing livestock diet quality. Among these, partial least squares regression (PLSR) is the most commonly employed model for predicting fecal, forage, and grain feed parameters, followed by modified partial least squares regression (MPLSR), a variant of PLS. This popularity can be attributed to several key advantages of PLSR. PLSR is highly effective in handling high-dimensional spectral data, multicollinearity, and noisy datasets, all of which are common in NIR analysis of fecal, forage, and grain feed samples. It reduces the dimensionality of the data while preserving the relationship between spectral information and the target variables, making it well-suited for these complex matrices [27], [63], [67]. Additionally, MPLSR is frequently used as an extension of PLSR to enhance robustness, especially in the presence of outliers or for achieving greater calibration accuracy in more heterogeneous samples [56], [6].

## 5 Discussion

NIRS is a widely used technology for analysing the quality of livestock. It offers several advantages over traditional analytical methods, including rapid and non-destructive analysis, low cost, and the ability to analyse multiple parameters simultaneously. In this review, we discuss the use of NIRS for analysing different aspects of livestock diet quality, including fecal analysis, forage analysis, and grain feed analysis.

### 5.1 Evolving applications of NIRS

The analysis of faeces plays a crucial role in assessing the health and nutritional status of animals. The composition of animal feces provides insights into the animal's diet quality and nutrient intake. Numerous studies have used NIRS to examine the composition of faeces, including protein, fat, and fibre content for estimating livestock diet quality. For instance, a study by Glasser et al. [39] used NIRS to predict the nutrient content of goat feces, achieving high accuracy in predicting crude protein and fibre content. Similarly, in [43], NIRS was used to analyse the chemical composition of cattle, goat, and sheep feces and found that it could accurately predict nitrogen and phosphorous.

Forage analysis is another critical aspect of livestock diet quality assessment. The nutritional value of forage is critical for ensuring optimal animal health and performance. NIRS has been used extensively to analyse the nutrient content of forage, including protein, fibre, and mineral content. Several studies have demonstrated the accuracy of NIRS for analysing forage quality. For example, NIRS was used to analyse the nutrient content of hay and straw samples, achieving high accuracy in predicting dry matter, crude protein, and fibre content [7]. Similarly, a study utilised NIRS to predict the nutritional value of chickpea straw, achieving high accuracy in predicting ash, protein, and fibre content [2].

NIRS has also been applied to assess the quality of grain feed. Analysing the nutrient content of grain feed is critical for ensuring optimal animal health and performance. Several studies have used NIRS to analyse the nutrient content of grain feed. For example, Modroño et al. [68] used NIRS to predict the nutrient content of crumbs and pellets, achieving high accuracy in predicting protein and starch content.

Over the past decade, the application of NIRS has significantly expanded from primarily laboratory-based analyses to more diverse and real-time settings, particularly with the advent of portable NIRS devices [78]. This progress has enabled on-site, immediate analysis of livestock feed, forage, and fecal matter, which has proven invaluable for making timely, data-driven decisions in precision livestock farming [4], [66], [31]. The growing adoption of NIRS technology has facilitated the optimisation of feed rations, improved livestock health, and enhanced production efficiency. Moreover, the integration of NIRS with advanced data processing techniques (e.g., regression) has opened new avenues for predicting animal performance and further refining livestock management practices, thereby contributing to more sustainable and productive agricultural systems [50].

In NIRS analysis, sample preparation plays a crucial role in obtaining consistent and accurate spectra, which directly impacts the quality of the calibration models. Proper sample preparation, such as grinding, drying, and homogenisation, ensures that the samples are uniform and representative, thus minimising variability due to physical characteristics like particle size or moisture content. Studies have shown that inadequate sample preparation can result in poor calibration performance and reduced model accuracy [79], [80]. The importance of standardised sample preparation procedures becomes even more critical when comparing data across different instruments or experimental locations.

External validation is a critical step in ensuring that NIRS calibration models are generalised to new, unseen datasets. While cross-validation provides insights into the internal consistency of a model using the available data, it does not fully test the model's ability to predict data from different experimental conditions or populations. External validation, which involves applying the model to an entirely independent dataset, is essential for assessing the robustness of the model and its practical applicability in real-world scenarios. Studies have demonstrated that models validated with external datasets tend to be more reliable and better suited for broader applications compared to those evaluated solely with cross-validation [81], [82]. In this review, several studies employed external validation techniques, thereby improving the reliability of their models for use in livestock diet quality analysis [66], [34], [61], [27]. In the following subsections, we discuss challenges and future works as well as the limitations of this study.

### 5.2 Challenges and future research directions

*Calibration models*  NIRS requires the development of robust and accurate calibration models specific to each parameter for livestock diet quality analysis. This process can be time-consuming and expensive, and requires access to many samples that represent the variability of the target population [66]. Also, the calibration models must be validated to ensure their accuracy and reliability. Developing new calibration models that account for the variability introduced by different factors such as animal breed, diet, and environmental conditions is an important area of future research.

*Sample preparation*  The accuracy of NIRS measurements can be affected by factors such as sample homogeneity, size, shape, and moisture content [32]. Therefore, it is crucial to prepare the samples carefully and consistently to obtain reliable results. However, the preparation process can be time-consuming and labour-intensive, especially for large-scale analyses. The automation and miniaturisation of NIRS equipment can reduce the cost and time required for sample preparation and data acquisition, making NIRS more practical for livestock producers.

*Interference*  NIRS measurements of feed are primarily reflective of the chemical composition of the sample being analysed. However, when NIRS is used in applications related to animal nutrition and health, the breed, age, sex, and diet of the animal can indirectly influence the composition of biological samples (such as faeces or tissue) analysed by NIRS [62]. For instance, different breeds or age groups may metabolise feed differently, leading to variations in nutrient absorption and fecal composition, which can introduce variability in NIRS measurements. Therefore, it is essential to include a diverse range of samples in calibration models to ensure they are robust and account for this variability. In addition, advanced data processing and machine learning techniques can be employed to reduce the impact of these interfering factors, potentially involving spectral preprocessing techniques to remove or reduce noise introduced by such variability. This approach can lead to more accurate and reliable NIRS measurements in livestock diet quality nutrition studies.

*Instrumentation*  The accuracy and reliability of NIRS measurements depend on the quality and performance of the equipment used. Therefore, it is essential to use high-quality instruments that are well-maintained and calibrated regularly. However, the cost of NIRS equipment can be prohibitive for small-scale livestock producers. The development of portable and miniaturised NIRS instruments can make it easier and more cost-effective to conduct on-site measurements in remote locations, such as on farms [51], [43]. Also, Developing NIRS instruments that can provide real-time analysis of livestock products can improve the speed and efficiency of quality control processes.

*Standardisation*  There is a lack of standardisation using NIRS for livestock diet quality analysis leading to variability and inconsistency in the results [32]. Therefore, it is crucial to develop standard protocols and guidelines for sample preparation, data acquisition, calibration, and validation to ensure the comparability and reproducibility of results. A major challenge in NIRS analysis is the lack of standardised reference data for the calibration and validation of instruments. The researchers could focus on developing standard reference databases for fecal, forage, and gain feed analysis that can be used to calibrate and validate NIRS instruments.

### 5.3 Limitations

In this review, the electronic database search for relevant research publications is one of the main limitations. We considered five electronic databases for this study and identified 54 articles using the search strings mentioned in the methodology section. As other databases were not considered, more publications might be available that have not been included in this review. In addition, the search keyword string might have prevented finding some relevant articles. To find as many pertinent articles as possible, we used a comprehensive search strategy that included both the primary search terms and their synonyms. Also, the selection of databases was carefully considered to align with the scope of this study.

Another potential limitation relates to the extraction of data from the reviewed papers, as there is a possibility of overlooking certain information during this process. We double-reviewed the analysed data to reduce any missing information. Although these limitations will open up opportunities for future review, they do not prevent the main objective of presenting an in-depth picture of the application of NIRS for the analysis of livestock diet quality.

Also, this review did not identify studies related to forage analysis that specifically focused on using NIRS for analysing Total Mixed Ration (TMR), despite its significance in the dairy and beef sectors. TMR is an essential feed component, as it blends forages, grains, proteins, and minerals into a single balanced ration. While literature exists on the use of NIRS for TMR analysis, it was not covered within the scope of this review. Future research or systematic reviews could address this gap by exploring NIRS applications for TMR analysis, particularly given the method's proven versatility in evaluating complex feed compositions.

Additionally, while this review focuses on summarising the application of NIRS technology as reported in published research articles, it is important to acknowledge that NIRS is also widely used in industry. The focus on academic research means that certain aspects of real-world industrial applications, such as practical implementation challenges, cost-effectiveness, and routine integration into livestock management practices, may not be fully covered. This distinction suggests that future studies could specifically explore the industrial use of NIRS to provide a more comprehensive understanding of its application beyond research settings.

## 6 Conclusion

This study conducts a comprehensive review on the applications of NIRS in analysing livestock diet quality, specifically addressing the key research questions identified in the introduction. Many articles were analysed, shedding light on the research significance of NIRS technology in the livestock industry. In this review, in response to the research questions, several critical findings have been identified, including the types of NIRS devices used, datasets used, methods for preprocessing spectral data, livestock diet quality characteristics studied, regression models applied, performance evaluation metrics used, and challenges associated with the implementation of NIRS in this domain. The review focused on three categories of livestock diet quality analysis, namely forage, fecal, and grain feed analysis. The results showed that NIRS technology could accurately predict various quality parameters in each category, demonstrating its versatility in livestock diet quality analysis. The study found most NIRS devices used in livestock diet quality analysis were either benchtop or portable and used reflectance spectra mode. Furthermore, the commonly used spectral data preprocessing methods were standard normal variate (SNV), detrend (DT), and multiplicative scatter correction (MSC). NIRS technology has the potential to revolutionise livestock diet quality analysis by providing fast, reliable, and non-destructive measurements of various quality parameters. However, there are still challenges that need to be addressed, such as interference from other factors, instrument standardisation, and the development of new analysis methods. Overall, this review answers the initial research questions and provides valuable insights into the current state and future potential of NIRS technology for livestock diet quality analysis.

## Funding statement

This project was supported by funding from 10.13039/100030797Food Agility CRC Ltd, funded under the Commonwealth Government CRC Program. The CRC Program supports industry-led collaborations between industry, researchers and the community.

## CRediT authorship contribution statement

**Md Ekramul Hossain:** Writing – original draft, Visualization, Methodology, Formal analysis, Data curation, Conceptualization. **Muhammad Ashad Kabir:** Writing – review & editing, Validation, Supervision, Methodology, Funding acquisition, Conceptualization. **Lihong Zheng:** Writing – review & editing, Validation, Supervision, Funding acquisition, Conceptualization. **David L. Swain:** Writing – review & editing, Funding acquisition, Conceptualization. **Shawn McGrath:** Writing – review & editing, Funding acquisition, Conceptualization. **Jonathan Medway:** Project administration, Funding acquisition.

## Declaration of Competing Interest

The authors declare the following financial interests/personal relationships which may be considered as potential competing interests: Muhammad Ashad Kabir is serving as an Associate Editor at Heliyon. The other authors declare that they have no known competing financial interests or personal relationships that could have appeared to influence the work reported in this paper.

## Data Availability

Not applicable.

## References

[1] Harris P.A., Nelson S., Carslake H.B., Argo C.M., Wolf R., Fabri F.B., Brolsma K.M., van Oostrum M.J., Ellis A.D. (2018). Comparison of nirs and wet chemistry methods for the nutritional analysis of haylages for horses. J. Equine Vet. Sci..

[2] Corson D., Waghorn G., Ulyatt M., Lee J. (1999). Proceedings of the New Zealand Grassland Association.

[3] Alemu T., Wamatu J., Tolera A., Beyan M., Eshete M., Alkhtib A., Rischkowsky B. (2021). Optimizing near infrared reflectance spectroscopy to predict nutritional quality of chickpea straw for livestock feeding. Animals.

[4] Rich B.T., Thomas D.B., Longnecker M.T., Tolleson D.R., Angerer J., de León A.A.P., Teel P.D. (2022). Bovine fecal chemistry changes with progression of southern cattle tick, rhipicephalus (boophilus) microplus (acari: Ixodidae) infestation. Vet. Parasitol..

[5] Núñez-Sánchez N., Carrion D., Blanco F.P., García V.D., Sigler A.G., Martínez-Marín A.L. (2016). Evaluation of botanical and chemical composition of sheep diet by using faecal near infrared spectroscopy. Anim. Feed Sci. Technol..

[6] Ikoyi A., Younge B. (2022). Faecal near-infrared reflectance spectroscopy profiling for the prediction of dietary nutritional characteristics for equines. Anim. Feed Sci. Technol..

[7] Foskolos A., Calsamiglia S., Chrenková M., Weisbjerg M., Albanell E. (2015). Prediction of rumen degradability parameters of a wide range of forages and non-forages by nirs. Animal.

[8] Huang J., Luo B., Cao Y., Li B., Qian M., Jia N., Zhao W. (2022). Fusion of thz-tds and nirs based detection of moisture content for cattle feed. Front. Phys..

[9] Lobos I., Moscoso C.J., Pavez P. (2019). Calibration models for the nutritional quality of fresh pastures by near-infrared reflectance spectroscopy. Cienc. Investig. Agrar., Rev. Latinoamer. Cienc. Agricultura.

[10] Evangelista C., Basiricò L., Bernabucci U. (2021). An overview on the use of near infrared spectroscopy (nirs) on farms for the management of dairy cows. Agriculture.

[11] Pu Y., Pérez-Marín D., O'Shea N., Garrido-Varo A. (2021). Recent advances in portable and handheld nir spectrometers and applications in milk, cheese and dairy powders. Foods.

[12] Kumaravelu C., Gopal A. (2015). 2015 IEEE Technological Innovation in ICT for Agriculture and Rural Development (TIAR).

[13] Chen L., Yang Z., Han L. (2013). A review on the use of near-infrared spectroscopy for analyzing feed protein materials. Appl. Spectrosc. Rev..

[14] Kho E.A., Fernandes J.N., Tilbrook A.J., Fox G.P., Sikulu-Lord M.T., Kotze A.C., Beasley A.M., James P.J., Tolleson D.R., Cozzolino D. (2022). State of the art and the future of fecal analysis using infrared spectroscopy. Appl. Spectrosc. Rev..

[15] Landau S., Glasser T., Dvash L. (2006). Monitoring nutrition in small ruminants with the aid of near infrared reflectance spectroscopy (nirs) technology: a review. Small Rumin. Res..

[16] Aromataris E., Pearson A. (2014). The systematic review: an overview. Am. J. Nurs..

[17] Kitchenham B., Charters S. (2007).

[18] Kitchenham B., Brereton O.P., Budgen D., Turner M., Bailey J., Linkman S. (2009). Systematic literature reviews in software engineering–a systematic literature review. Inf. Softw. Technol..

[19] Wohlin C. (2014). Proceedings of the 18th International Conference on Evaluation and Assessment in Software Engineering.

[20] Siesler H.W., Kawata S., Heise H.M., Ozaki Y. (2008).

[21] Beć K.B., Grabska J., Huck C.W. (2021). Principles and applications of miniaturized near-infrared (nir) spectrometers. Chemistry.

[22] Zhao Y., Chen Y. (2023). Global patterns of agricultural investment and food security: evidence from the fdi markets database. Foods.

[23] Heisey P.W., Fuglie K.O. (2018).

[24] Hart J.F., Mayda C. (1998). The industrialization of livestock production in the United States. Southeastern Geographer.

[25] Bai Z., Ma W., Ma L., Velthof G.L., Wei Z., Havlík P., Oenema O., Lee M.R., Zhang F. (2018). China's livestock transition: driving forces, impacts, and consequences. Sci. Adv..

[26] Rauw W.M., Gómez Izquierdo E., Torres O., García Gil M., de Miguel Beascoechea E., Rey Benayas J.M., Gomez-Raya L. (2023). Future farming: protein production for livestock feed in the EU. Sustain. Earth Rev..

[27] Kneebone D., Dryden G.M. (2014). Prediction of diet quality for sheep from faecal characteristics: comparison of near-infrared spectroscopy and conventional chemistry predictive models. Anim. Reprod. Sci..

[28] Dillon E.J., Hennessy T., Hynes S. (2010). Assessing the sustainability of Irish agriculture. Int. J. Agric. Sustain..

[29] Wajizah S., Munawar A. (2018).

[30] Ritchie H., Rosado P., Roser M. (2017).

[31] Ryckewaert M., Chaix G., Héran D., Zgouz A., Bendoula R. (2022). Evaluation of a combination of nir micro-spectrometers to predict chemical properties of sugarcane forage using a multi-block approach. Biosyst. Eng..

[32] Reddersen B., Fricke T., Wachendorf M. (2013). Effects of sample preparation and measurement standardization on the nirs calibration quality of nitrogen, ash and ndfom content in extensive experimental grassland biomass. Anim. Feed Sci. Technol..

[33] Zhang Y., Chen H., Chen W., Xu L., Li C., Feng Q. (2021). Near infrared feature waveband selection for fishmeal quality assessment by frequency adaptive binary differential evolution. Chemom. Intell. Lab. Syst..

[34] Lahart B., McParland S., Kennedy E., Boland T., Condon T., Williams M., Galvin N., McCarthy B., Buckley F. (2019). Predicting the dry matter intake of grazing dairy cows using infrared reflectance spectroscopy analysis. J. Dairy Sci..

[35] Assouma M.H., Lecomte P., Hiernaux P., Ickowicz A., Corniaux C., Decruyenaere V., Diarra A., Vayssières J. (2018). How to better account for livestock diversity and fodder seasonality in assessing the fodder intake of livestock grazing semi-arid sub-Saharan Africa rangelands. Livest. Sci..

[36] Bastianelli D., Bonnal L., Jaguelin-Peyraud Y., Noblet J. (2015). Predicting feed digestibility from nirs analysis of pig faeces. Animal.

[37] Hassoun P., Bastianelli D., Autran P., Bocquier F. (2014). Polyethylene glycol compared with ytterbium oxide as a total faecal output marker to predict organic matter intake of dairy ewes fed indoors or at pasture. Animal.

[38] Hassoun P., Viudes G., Autran P., Bastianelli D., Bocquier F. (2013). A method for estimating dry forage intake by sheep using polyethylene glycol as a faecal marker measured with nirs. Animal.

[39] Glasser T., Landau S., Ungar E., Perevolotsky A., Dvash L., Muklada H., Kababya D., Walker J. (2012). Foraging selectivity of three goat breeds in a Mediterranean shrubland. Small Rumin. Res..

[40] Čupić S., García A.J., Holá M., Ceacero F. (2021). Evaluation of factors inducing variability of faecal nutrients in captive red deer under variable demands. Sci. Rep..

[41] Nirea K.G., Pérez de Nanclares M., Skugor A., Afseth N.K., Meuwissen T.H., Hansen J.Ø., Mydland L.T., Øverland M. (2018). Assessment of fecal near-infrared spectroscopy to predict feces chemical composition and apparent total-tract digestibility of nutrients in pigs. J. Anim. Sci..

[42] Tolleson D., Schafer D. (2014). Application of fecal near-infrared spectroscopy and nutritional balance software to monitor diet quality and body condition in beef cows grazing Arizona rangeland. J. Anim. Sci..

[43] Tolleson D., Angerer J. (2021). The application of near infrared spectroscopy to predict faecal nitrogen and phosphorus in multiple ruminant herbivore species. Rangeland J..

[44] González L., Charmley E., Henry B. (2014). Modelling methane emissions from remotely collected liveweight data and faecal near-infrared spectroscopy in beef cattle. Anim. Reprod. Sci..

[45] Noel S.J., Jørgensen H.J.H., Knudsen K.E.B. (2021). Prediction of protein and amino acid composition and digestibility in individual feedstuffs and mixed diets for pigs using near-infrared spectroscopy. Anim. Nutr..

[46] Landau S., Dvash L., Roudman M., Muklada H., Barkai D., Yehuda Y., Ungar E. (2016). Faecal near-ir spectroscopy to determine the nutritional value of diets consumed by beef cattle in East Mediterranean rangelands. Animal.

[47] Ryan S.J., Cross P.C., Winnie J., Hay C., Bowers J., Getz W.M. (2012). The utility of normalized difference vegetation index for predicting African buffalo forage quality. J. Wildl. Manag..

[48] Decruyenaere V., Froidmont E., Bartiaux-Thill N., Buldgen A., Stilmant D. (2012). Faecal near-infrared reflectance spectroscopy (nirs) compared with other techniques for estimating the in vivo digestibility and dry matter intake of lactating grazing dairy cows. Anim. Feed Sci. Technol..

[49] Righi F., Simoni M., Visentin G., Manuelian C.L., Currò S., Quarantelli A., De Marchi M. (2017). The use of near infrared spectroscopy to predict faecal indigestible and digestible fibre fractions in lactating dairy cattle. Livest. Sci..

[50] Soldado A., Fearn T., Martínez-Fernández A., De La Roza-Delgado B. (2013). The transfer of nir calibrations for undried grass silage from the laboratory to on-site instruments: comparison of two approaches. Talanta.

[51] Berzaghi P., Cherney J.H., Casler M.D. (2021). Prediction performance of portable near infrared reflectance instruments using preprocessed dried, ground forage samples. Comput. Electron. Agric..

[52] Oluk A.C., Yucel H., Bilgin F.D., Serbester U. (2022). Estimation of forage quality by near infrared reflectance spectroscopy in dallisgrass, paspalum dilatatum, poir. J. Near Infrared Spectrosc..

[53] Acosta J., Castillo M., Hodge G. (2020). Comparison of benchtop and handheld near-infrared spectroscopy devices to determine forage nutritive value. Crop Sci..

[54] Monrroy M., Gutiérrez D., Miranda M., Hernández K., Renán García J. (2017). Determination of brachiaria spp. forage quality by near-infrared spectroscopy and partial least squares regression. J. Chil. Chem. Soc..

[55] Yang Z., Nie G., Pan L., Zhang Y., Huang L., Ma X., Zhang X. (2017). Development and validation of near-infrared spectroscopy for the prediction of forage quality parameters in lolium multiflorum. PeerJ.

[56] Asekova S., Han S.-I., Choi H.-J., Park S.-J., Shin D.-H., Kwon C.-H., Shannon J.G., Lee J.D. (2016). Determination of forage quality by near-infrared reflectance spectroscopy in soybean. Turk. J. Agric. For..

[57] Norman H.C., Hulm E., Humphries A.W., Hughes S.J., Vercoe P.E. (2020). Broad near-infrared spectroscopy calibrations can predict the nutritional value of > 100 forage species within the Australian feedbase. Anim. Reprod. Sci..

[58] Murphy D.J., O'Brien B., O'Donovan M., Condon T., Murphy M.D. (2022). A near infrared spectroscopy calibration for the prediction of fresh grass quality on Irish pastures. Inf. Process. Agric..

[59] Rego G., Ferrero F., Valledor M., Campo J.C., Forcada S., Royo L.J., Soldado A. (2020). A portable iot nir spectroscopic system to analyze the quality of dairy farm forage. Comput. Electron. Agric..

[60] Almanjahie I.M., Ahmad I., Chikr Elmezouar Z., Laksaci A. (2019). Modern statistical analysis of forage quality assessment with nir spectroscopy. Appl. Ecol. Environ. Res..

[61] Baath G.S., Baath H.K., Gowda P.H., Thomas J.P., Northup B.K., Rao S.C., Singh H. (2020). Predicting forage quality of warm-season legumes by near infrared spectroscopy coupled with machine learning techniques. Sensors.

[62] Parrini S., Acciaioli A., Franci O., Pugliese C., Bozzi R. (2019). Near infrared spectroscopy technology for prediction of chemical composition of natural fresh pastures. J. Appl. Anim. Res..

[63] Hetta M., Mussadiq Z., Wallsten J., Halling M., Swensson C., Geladi P. (2017). Prediction of nutritive values, morphology and agronomic characteristics in forage maize using two applications of nirs spectrometry. Acta Agric. Scand., B Soil Plant. Sci..

[64] Lobos I., Gou P., Hube S., Saldaña R., Alfaro M. (2013). Evaluation of potential nirs to predict pastures nutritive value. J. Soil Sci. Plant Nutr..

[65] Despal D., Sari L., Chandra R., Zahera R., Permana I., Abdullah L. (2020). Prediction accuracy improvement of Indonesian dairy cattle fiber feed compositions using near-infrared reflectance spectroscopy local database. Trop. Anim. Sci. J..

[66] Ferreira L., Machado N., Gouvinhas I., Santos S., Celaya R., Rodrigues M., Barros A. (2022). Application of Fourier transform infrared spectroscopy (ftir) techniques in the mid-ir (mir) and near-ir (nir) spectroscopy to determine n-alkane and long-chain alcohol contents in plant species and faecal samples. Spectrochim. Acta, Part A, Mol. Biomol. Spectrosc..

[67] Wajizah S., Munawar A.A. (2020). Near infrared spectroscopy (nirs) data analysis for a rapid and simultaneous prediction of feed nutritive parameters. Data Brief.

[68] Modroño S., Soldado A., Martínez-Fernández A., de la Roza-Delgado B. (2017). Handheld nirs sensors for routine compound feed quality control: real time analysis and field monitoring. Talanta.

[69] Haughey S.A., Graham S.F., Cancouët E., Elliott C.T. (2013). The application of near-infrared reflectance spectroscopy (nirs) to detect melamine adulteration of soya bean meal. Food Chem..

[70] Graham S.F., Haughey S.A., Ervin R.M., Cancouët E., Bell S., Elliott C.T. (2012). The application of near-infrared (nir) and Raman spectroscopy to detect adulteration of oil used in animal feed production. Food Chem..

[71] Wajizah S., Zulfahrizal Z., Munnawar A. (2022).

[72] Wajizah S., Zulfahrizal Z. (2021).

[73] Samadi S., Wajizah S., Munawar A. (2018). Rapid and simultaneous determination of feed nutritive values by means of near infrared spectroscopy. Trop. Anim. Sci. J..

[74] Hoffmann D., Bruggerb D., Windischb W., Thurnera S. (2017). Calibration model for a near infrared spectroscopy (nirs) system to control feed quality of soy cake based on feed value assessments in-vitro. Chem. Eng..

[75] Hu J., Li J., Pan L., Piao X., Sui L., Xie G., Zhang S., Zhang L., Wang J. (2019). Rapid determination of the content of digestible energy and metabolizable energy in sorghum fed to growing pigs by near-infrared reflectance spectroscopy. J. Anim. Sci..

[76] Reeves J.B. (2000).

[77] Heise H.M., Schulenburg R. (2022). Molecular and Laser Spectroscopy.

[78] Vincent B., Dardenne P. (2021). Application of nir in agriculture, near-infrared spectroscopy. Theory Spectr. Anal. Instr. Appl..

[79] Murray I., Cowe I. (2004). Sample preparation. Near Infrared Spectrosc. Agric..

[80] Ikoyi A., Younge B.A. (2020). Influence of forage particle size and residual moisture on near infrared reflectance spectroscopy (nirs) calibration accuracy for macro-mineral determination. Anim. Feed Sci. Technol..

[81] Shenk J., Westerhaus M. (1991). Population definition, sample selection, and calibration procedures for near infrared reflectance spectroscopy. Crop Sci..

[82] Williams P., Norris K. (1987).

